# Organic Acids Secreted by *Lactobacillus* spp. Isolated from Urine and Their Antimicrobial Activity against Uropathogenic *Proteus mirabilis*

**DOI:** 10.3390/molecules27175557

**Published:** 2022-08-29

**Authors:** Dominika Szczerbiec, Justyna Piechocka, Rafał Głowacki, Agnieszka Torzewska

**Affiliations:** 1 Department of Biology of Bacteria, Faculty of Biology and Environmental Protection, University of Lodz, Banacha 12/16, 90-237 Lodz, Poland; 2 Department of Environmental Chemistry, Faculty of Chemistry, University of Lodz, Pomorska 163/165, 90-236 Lodz, Poland

**Keywords:** organic acids, *Lactobacillus*, *Proteus mirabilis*, microbiota, antibacterial activity

## Abstract

The natural microbiota of the urinary tract includes *Lactobacillus* spp., which secrete molecules with antimicrobial properties and have antagonistic activity against many pathogens. This paper focuses on the antibacterial effect of *Lactobacillus* strains isolated from urine against clinical strains of *Proteus mirabilis* isolated from kidney stones and from urine with coexisting urolithiasis. The study involved analyzing the main antimicrobial molecules secreted by *Lactobacillus.* In order to indicate which agent had the strongest antimicrobial effect, the supernatants were made alkaline and treated with catalase and high temperature. Both treated and untreated supernatants were analyzed for their activity. Exposing uropathogens to all untreated cell-free supernatants of *Lactobacillus* significantly reduced their growth, and it was established that these properties were related to organic acid secretion by these strains. Using LC–MS/MS and spectrophotometric techniques, lactic, citric, and succinic acids were determined qualitatively and quantitatively. The influence of these acids on the *P. mirabilis* growth and biofilm formation and their influence on membrane permeability were also investigated. The results indicate that organic acids secreted by *Lactobacillus* strains have a high antibacterial potential and could be used as novel agents in the treatment of urinary tract infections caused by *P. mirabilis*.

## 1. Introduction

The genus *Lactobacillus* belongs to the group of lactic acid bacteria (LAB), which consists of a large and diverse spectrum of bacterial species. In general, these bacteria are nonpathogenic, microaerophilic, catalase-negative, Gram-positive rods [1]. It is well known that *Lactobacillus* spp. are part of the normal human microbiota, mainly of the healthy female genital tract [2,3]. Recently, these bacteria have been shown to be an important component of the urinary tract microbiota. The urinary tract used to be considered a sterile environment. However, molecular biology techniques and novel culture methods have allowed isolating and characterizing healthy human urinary tract microbiota [4,5]. The urinary tract and urine are the habitat of microorganisms, mainly *Lactobacillus* and *Streptococcus*, in addition to microbes of the genera *Gardnerella, Prevotella, Corynebacterium*, and *Sneathia*, playing a protective role against pathogens [6,7]. The microbiota of the genitals and urinary tract consist of highly similar strains of health-associated commensal bacteria and, as reported in the literature, the most common *Lactobacillus* strains isolated from urine and bladder belonging to *L. crispatus, L. iners*, *L. gasseri*, and *L. jensenii* species [5,7].

*Lactobacillus* bacteria are believed to have probiotic properties, and they modulate the host immunity. Their antimicrobial activity against many pathogens, including those causing urinary tract infections (UTIs), has been comprehensively demonstrated. *Lactobacillus* species produce antibacterial compounds such as biosurfactants, bacteriocins, hydrogen peroxide, and organic acids [8,9]. The production of bacteriocins by *Lactobacillus* and their antibacterial properties against many pathogenic bacteria have been widely explored [10,11,12]. Similarly, their ability to produce hydrogen peroxide and their impact on pathogen growth inhibition have also been described [13,14]. However, in many cases, the antimicrobial activity of *Lactobacillus* strains is associated with organic acid production. The most common organic acids found in the fermentation broth include lactic acid, citric acid, acetic acid, butyric acid, and propionic acid in the case of *L. plantarum* and *L. crispatus* strains [15,16]. Despite the fact that, recently, more attention has been paid to the microbiota of the urinary tract, there are not enough reports regarding strains isolated from the urinary tract and interactions between the natural microbiota of the urinary tract and uropathogens. 

One of the pathogens frequently causing urinary tract infections (UTIs), especially those in long-term catheterization patients, is *Proteus mirabilis. Proteus* spp. belong to the family Morganellaceae, widely existing both in the natural environment and as part of the human microbiota, predominantly as a commensal of the gastrointestinal tracts of human and animals. *P. mirabilis* is well known due to its virulence factors such as urease, an enzyme that can hydrolyze urea into ammonia and carbon dioxide, which raises the urine pH to facilitate the formation of kidney stones, as well as swarming motility and the presence of fimbriae and flagella. Due to the difficulties in combating *Proteus* infections, there is a risk of permanent renal damage and urinary stone formation, which may progress to bacteremia and sepsis [17,18]. The treatment of patients with UTIs is long-term and often ineffective, consisting mainly of the use of antibiotics. It should be emphasized that UTIs are at the forefront of the antibiotic resistance problem. A common problem is also the formation of a bacterial biofilm on urinary catheters by urease-positive *P. mirabilis*. Accumulation of calcium and magnesium ions may occur in the biofilm, which, in combination with the activity of urease, causes the formation of crystals and incrustation of the urinary catheter, possibly leading to its blockage [19]. Thus, there is an urgent need to develop alternative therapeutics for the treatment of UTIs. The natural microbiota of the urinary tract can be a source of strains antagonistic to uropathogenic bacteria. Those strains or the antibacterial substances secreted by them can be used to treat or prevent UTIs [20].

The present paper was designed to expand the knowledge on the interactions between the existing members of the beneficial host urinary tract microbiota and uropathogens. The study focused on the substances with antibacterial properties excreted by lactobacilli and their characteristics. The purpose of this work was to determine the antibacterial effect of *Lactobacillus* species isolated from urine on one of the most common UTI etiological agents-*P. mirabilis*.

## 2. Results

### 2.1. Antibiotic Susceptibility Profile of Proteus Mirabilis Strains

An antibiotic susceptibility test was carried out on eight strains of *P. mirabilis* with 15 antibiotics used in the treatment of patients with urinary tract infections including penicillins, cephalosporins, carbapenems, monobactams, fluoroquinolones, aminoglycosides, tetracyclines, and nitrofurans. As shown in Table 1, most of the tested strains were resistant to ampicillin, ampicillin/sulbactam, cefotaxime, gentamicin, and nitrofurantoin. Two of the eight tested strains (*P. mirabilis* 608/221 and 1090) were multidrug-resistant bacteria. The most effective antibiotics were imipenem and norfloxacin, to which all tested strains were sensitive.

### 2.2. Antibacterial Effect of Lactobacillus CFSs on Pathogenic P. mirabilis Strains

**Figure 1:**
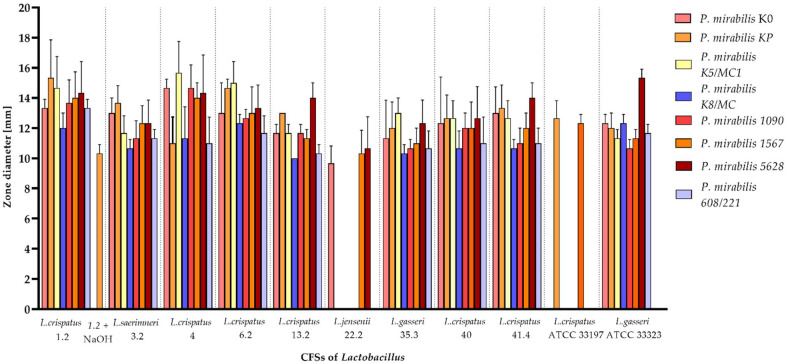
Zone of inhibition of *Lactobacillus* strains against *P. mirabilis* according to well diffusion assay. The data of CFSs treated with NaOH (excluding *L. crispatus* 1.2) are not shown due to the lack of *Proteus* growth inhibition. The results are presented as the mean ± standard deviation (SD) of three experiments. All the results are statistically significant (*p* < 0.05), according to *U Mann–Whitney* test.

*Lactobacillus* species exhibit certain antimicrobial mechanisms, and one of them is the production of antimicrobial substances including organic acids, hydrogen peroxide, and bacteriocins. In the well diffusion assay, the influence of cell-free supernatants (CFSs) of *Lactobacillus* on *P. mirabilis* growth inhibition was assessed. As presented in Figure 1, untreated CFSs of all nine *Lactobacillus* isolates and reference strains significantly inhibited the growth of most *P. mirabilis* bacteria (all *p* < 0.05). They showed average growth inhibition at the level 10–15 mm. However, the degree of antagonism varied among the *Lactobacillus* strains. *L. crispatus* (1.2; 4; 6.2) was the most effective, while *L. jensenii* (22.2) and the reference strain of *L. crispatus* were the weakest. As shown in Figure 1, only *L. jensenii* 22.2 and *L. crispatus* ATCC 33197 did not exhibit antibacterial activity against all of the pathogenic bacteria. It is worth noting that, in most cases, the antimicrobial effect was not observed in supernatants treated with 1 M NaOH. Only the CFS of *L. crispatus* 1.2 neutralized to pH 6.0 inhibited the growth of *P. mirabilis* KP.

Similarly, in the broth microdilution assay (Table 2), untreated CFSs showed a significantly strong inhibition effect on the growth of *P. mirabilis* (all *p* < 0.05). The greatest activity against pathogenic strains, with the percentage range of growth inhibition 62–99%, was observed in nine isolates (*L. crispatus* 1.2, 4, 13.2, 40, 41.4, *L. saerimneri* 3.2, *L. gasseri* 35.3, and two references strains *L.gasseri* ATCC 33323 and *L.crispatus* ATCC 33197), in contrast to the supernatants of *L. crispatus* 6.2 and *L. jensenii* 22.2, which showed the lowest percentage of *P. mirabilis* growth inhibition (62–79%).

**Figure 2:**
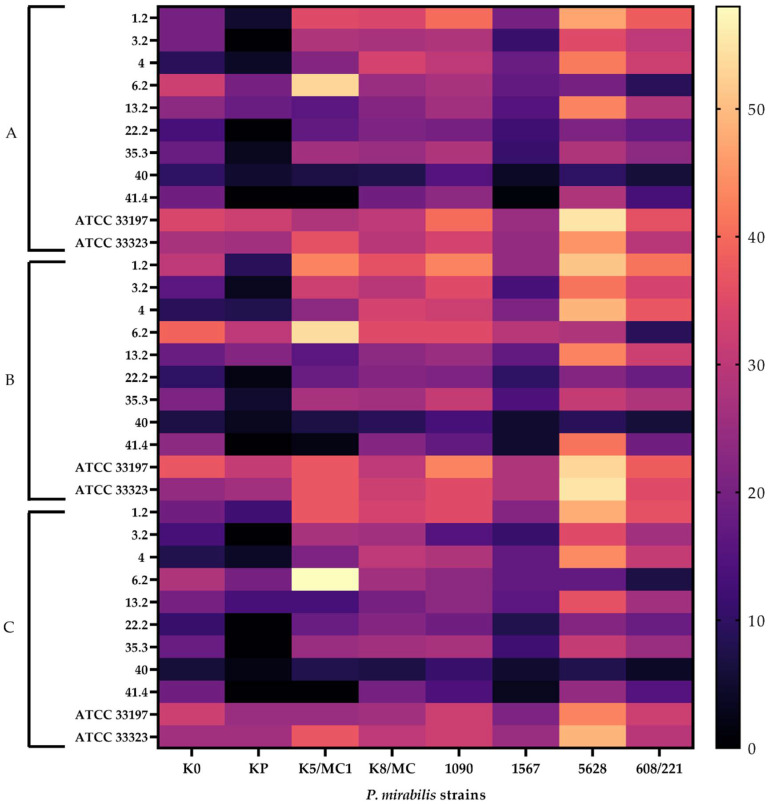
Heatmap of the *P. mirabilis* strain growth inhibition by cell-free *Lactobacillus* supernatants treated with different factors, expressed as percentages. (**A**) Supernatants neutralized to pH 6.0 and heated. (**B**) Supernatants only neutralized to pH 6.0. (**C**) Supernatants neutralized to pH 6.0 and treated with catalase. The results are presented as the mean ± standard deviation (SD) of three experiments, according to *Cochran’s Q test*.

As already mentioned, untreated supernatants exhibited the highest antimicrobial activity. On the other hand, the other three supernatants (treated with catalase, neutralized to pH 6.0, and heated) also showed an inhibitory effect, albeit with up to twofold lower efficiency, with a maximum value of 55% (Figure 2). The results of growth inhibition of *P. mirabilis* strains were at a similar level across trials with supernatants treated with different factors. However, when comparing the data obtained for individual *P. mirabilis* strains with different supernatants of *Lactobacillus* strains, differences were visible. The *P. mirabilis* KP strain exhibited the lowest inhibition, while strain 5628 showed the highest percentage growth inhibition by all tested supernatants. Low antibacterial properties of *L. crispatus* 40, treated with all factors, against all *Proteus* strains were also observed. 

All these results indicate that organic acids secreted by *Lactobacillus* had the strongest impact and were the main factors inhibiting the growth of *Proteus*. However, it is worth noting that other substances produced by lactobacilli such as hydrogen peroxide and bacteriocins may also influence the antibacterial effect of *Lactobacillus* although to a lesser extent than organic acids.

### 2.3. Profile of Organic Acids Produced by the Tested Lactobacillus Strains

**Figure 3:**
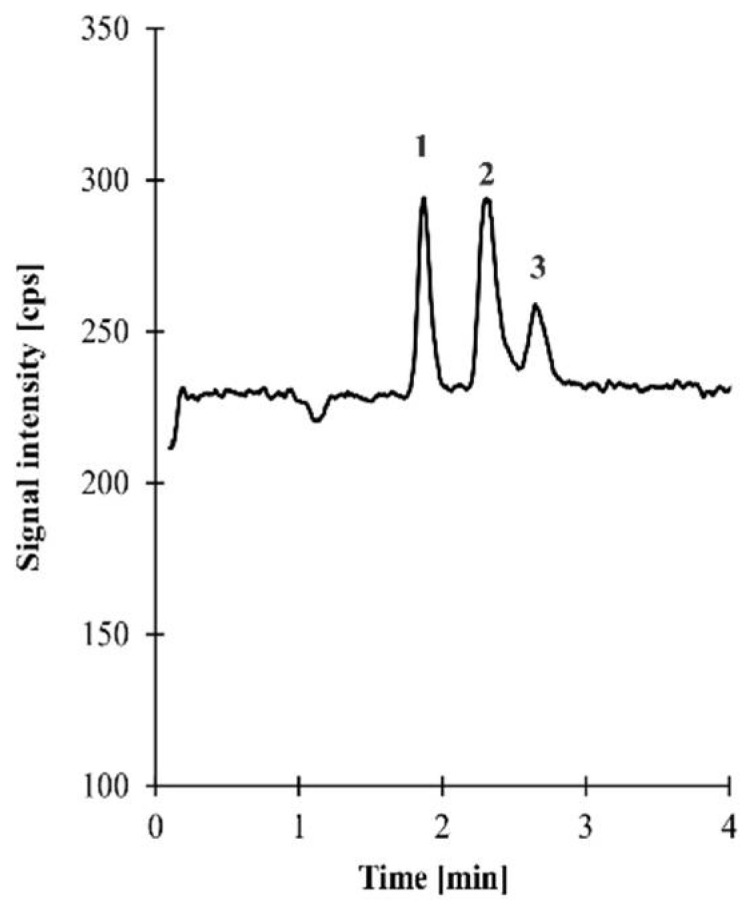
A representative total ion chromatogram of sample 13.2 prepared according to the procedure described in Section 4.5. Chromatographic conditions were as described in Section 4.5. Peaks: 1-lactic acid, 2-citric acid, 3-succinic acid (for assay details, see Supplementary Materials).

*Lactobacillus* spp. produce a variety of organic acids with antimicrobial properties. In our study, lactic, citric, succinic, acetic, propionic, and butyric acids were selected for determination of their presence. Qualitative analyses showed that all of the tested *Lactobacillus* strains produced lactic and succinic acids. Moreover, in the case of *L. crispatus* 13.2, *L. crispatus* 40, and the reference strain *L. gasseri*, the presence of citric acid was detected (Figure 3). Quantitative analyses exhibited the concentrations of these organic acids shown in Table 3. The organic acids produced by *Lactobacillus* strains were mainly lactic and succinic acids. The highest concentration of lactic acid was observed in *L. crispatus* 1.2 (25.09 mM) and *L. saerimneri* 3.2 (21.46 mM). The highest amount of citric acid was produced by *L. crispatus* 40, and *L. jensenii* 22.2 was characterized by the highest concentration of succinic acid (52.11 mM).

### 2.4. The Effect of Selected Organic Acids on P. mirabilis Cells

**Figure 4:**
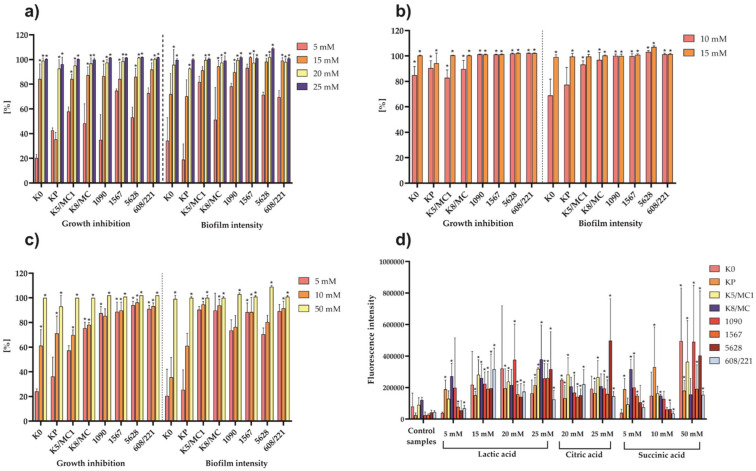
The percentage of *P. mirabilis* strain growth and biofilm formation inhibition by lactic acid (**a**), citric acid (**b**), and succinic acid (**c**). The results are presented as the mean ± standard deviation (SD) of three experiments. * *p* < 0.05 for comparison of bacterial growth in the presence of tested organic acids vs. *P. mirabilis* culture without organic acids, according to *Cochran’s Q* test. (**d**) The fluorescence intensity of *P. mirabilis* in the control culture (without acids) vs. that in the culture incubated with different concentration of organic acids. The results are presented as the mean ± standard deviation (SD) of four experiments. * *p* < 0.05 for comparison of fluorescence intensity of *P. mirabilis* treated with acids vs. pure *P. mirabilis* culture, according to *U Mann–Whitney test*.

The effect of selected concentrations of lactic, citric, and succinic acids produced by tested *Lactobacillus* strains on growth inhibition and biofilm formation by *P. mirabilis* strains was tested. The concentrations were selected on the basis of quantitative HPLC results and spectrophotometric methods. A decrease in *P. mirabilis* growth and biofilm formation was observed in samples with the highest concentrations of organic acid solutions. As shown in Figure 4, lactic acid from a concentration of 15 mM inhibited the growth of all *P. mirabilis* strains (except *P. mirabilis* KP). At this concentration, lactic acid was also found to inhibit the biofilm formation of most of the strains tested. Only K0, KP, and K5/MC1 *P. mirabilis* strains showed a greater tolerance range to the action of lactic acid, and, in these cases, the inhibition of biofilm formation was observed at a concentration of 20 mM. Citric acid was distinguished as a highly effective agent in inhibiting the growth of the tested uropathogens. Its concentration of 10 mM significantly inhibited the biofilm formation of al strains except K0 and KP. Succinic acid also showed an antibacterial effect against *P. mirabilis*. The most effective concentration was 50 mM, which significantly inhibited the growth and biofilm formation of all pathogenic strains. These results indicate that citric acid was the most effective against *P. mirabilis* strains.

One of the proposed mechanisms of the combatant action of organic acids on microorganisms is their ability to cross the cell membrane, which in most cases leads to bacterial cell death. To evaluate the condition of the *P. mirabilis* cell membrane in the presence of tested organic acids, an experiment with propidium iodide, which passes through the damaged cell membrane and binds to DNA, was performed. The U Mann-Whitney test showed significant differences between the fluorescence intensity in the control samples (untreated *P. mirabilis* cells) and samples incubated with acids (Figure 4). In most cases, the observed effect increased along with the increasing lactic, citric, and succinic acids concentrations. However, lactic acid caused significant damage to the *P. mirabilis* cell membrane from a concentration of 15 mM, while citric acid showed this effect from 10 mM, and, in the case of succinic acid, a significant change in fluorescence intensity was observed at 50 mM.

The above results indicate that the mechanisms of action of tested organic acids, which exhibited a strong antibacterial effect, involve penetrating the membrane of the pathogen and disrupting the cell function.

## 3. Discussion

The present study reports on the antibacterial effects of cell-free supernatants from *Lactobacillus* strains (*L. crispatus, L. gasseri, L. jensenii,* and *L. saerimneri*) isolated from human urine. The focus was on the characteristics of the antimicrobial effect of substances secreted by *Lactobacillus* on clinical strains of *P. mirabilis* with the indication of the factor with the highest activity. On the basis of many studies, we expected that natural microbiota of the urinary tract which include *Lactobacillus* strains would positively affect the condition of the urinary system and have an inhibitory effect on the development of *P. mirabilis* infections [21,22,23]. Dysbiosis of the natural microbiota of the urinary tract is related to the development of infections. Under normal conditions, the genitourinary microbiota plays a protective role against urinary tract infections [24]. Lewis et al. [25] showed that women with low levels of *Lactobacillus* are more commonly colonized with pathogens than those with diverse microbiota, which naturally decreases the risk of UTI development. However, changes in the natural microbiota of the urogenital system can be caused by many factors and are associated with age [26], medical treatment [7], diet, or diabetes [27]. Although there are many scientific reports on the antibacterial properties of *Lactobacillus* against pathogens, these data mainly concern strains isolated from the genital tract or food [8,28,29]. The innovativeness of these studies is based on the understanding of the interactions between microorganisms constantly present in the urinary tract and uropathogens. 

Our work focused on the evaluation of the antimicrobial effects of *Lactobacillus* agents against *P. mirabilis* due to the fact that this pathogen causes 1–10% of all urinary tract infections, the treatment of which is difficult and requires antibiotic therapy. Treatment for acute uncomplicated cystitis caused by *P. mirabilis* involves a 3 day course of double-strength trimethoprim/sulfamethoxazole (SXT). Alternative antibiotic therapies include the use of fluoroquinolone, nitrofurantoin, or fosfomycin [30]. However, *Proteus* spp. have developed resistance to several classes of antibiotics, which was also confirmed by our results (Table 1). A high percentage of the isolates were found to be resistant to ampicillin, ampicillin/sulbactam, cefotaxime, gentamicin, and nitrofurantoin. In the literature data, there are many reports about bacterial resistance to SXT, β-lactams, fluoroquinolones, nitrofurantoin, fosfomycin, aminoglycosides, tetracyclines, and sulfonamides [30,31,32,33]. Due to this phenomenon, there is a need to explore other effective and alternative drugs for the treatment of UTIs and their complications. 

The tested *Lactobacillus* strains isolated from the urine of healthy people and two reference strains showed antibacterial properties against *P. mirabilis* with varying degrees of intensity. Many researchers also focused on this aspect of research involving *Proteus* spp. Shaaban et al. [34] reported the antimicrobial, anti-adherence, and anti-biofilm activities of two probiotic *L. casei* and *L. reuteri* strains against multidrug-resistant *P. mirabilis* strains. In the course of our research, we showed that various compounds secreted extracellularly were responsible for these properties. Nevertheless, organic acids showed the highest percentage inhibition of *P. mirabilis* growth, which was confirmed by the well diffusion assay and broth microdilution method. A similar effect of organic acids was observed in the case of *Lactobacillus* supernatants against various pathogens such as *S. aureus*, *S.* Typhimurium, *E. coli, E. faecalis, C. difficile*, and *A. acidoterrestris* [35,36,37]. In this study, we identified the organic acids which played a key role in bacterial growth inhibition. Three common organic acids (lactic acid, citric acid, and succinic acid) were detected. There are many papers about the antimicrobial activity of organic acids secreted by *Lactobacillus*, but they mostly concern the species of *L. plantarum* and species isolated from food [15,38] or their area of interest is mainly lactic acid [39,40]. Hence, determining the concentrations of other acids exhibiting antibacterial properties, secreted by microorganisms of the urinary tract, constitutes a significant supplement to the current knowledge. It is worth mentioning that there is scientific evidence for the antibacterial effect of citric, mandelic, malic, propionic, lactic, hippuric, benzoic, and pyruvic acids against *P. mirabilis* and other pathogens [41].

Urinary tract infections are also associated with biofilm formation by *P. mirabilis*. As many as 48% of the isolated *Proteus* species have the ability to form a biofilm [42]. The formation of a bacterial biofilm is particularly dangerous because it promotes the growth of microorganisms resistant to antibiotics and many other factors, leading to the progression of infections and development of chronic diseases. It is also responsible for blocking urinary catheters by crystalline deposits [34,43]. It has been proven that *Lactobacillus* cell-free supernatants have excellent inhibitory abilities against the growth and biofilm formation of various bacteria species [44,45]. Ray Mohapatra et al. [46] reported that *L. plantarum* bacteriocin exhibited a broad-spectrum activity against Gram-positive and Gram-negative bacteria and had an ability to inhibit the formation of catheter-associated biofilm by *Pseudomonas aeruginosa* and *Staphylococcus aureus*. However, our work was concerned with the effect of the organic acids secreted by *Lactobacillus* strains and their action on *P. mirabilis* biofilm and growth. Acid concentrations were selected on the basis of their determination by HPLC and spectrophotometric methods in *Lactobacillus* cell-free supernatants. These strains differed from each other in terms of the proportions of secreted organic acids, which had an impact on their antimicrobial capacity. Over the course of the experiments, we found that organic acids in different concentrations significantly reduced the bacterial growth and biofilm formation (Figure 4). It turned out that citric acid was highly effective, and lactic acid at a concentration of 20–25 mM exhibited up to 100% effectiveness. Burns et al. [41] examined the antibacterial effect of some organic acids including citric and lactic acids against *P. mirabilis* growth and biofilm formation. Similarly to our results, a higher concentration of organic acids led to a greater effectiveness of growth inhibition and biofilm formation. In their work citric acid significantly inhibited the formation of *P. mirabilis* biofilm at a concentration of 7.8 mM, which is a result similar to our observations. In our study, we also proved that the inhibition of *P. mirabilis* growth and biofilm formation depended on the molar concentration of organic acids, and we demonstrated the potential of organic acids to inhibit biofilm formation on various surfaces, e.g., on catheters. 

Undissociated forms of organic acids easily penetrate the lipid membrane of the bacterial cell, before dissociating into anions and protons. Protons of these organic acids lead to cytoplasmic acidification and disruption of certain cell functions. On the other hand, accumulation of anions also results in disruption of metabolic functions, increased osmotic pressure, and cell death [47]. Due to this fact, we assessed *P. mirabilis* viability as a function of the membrane permeability. The results confirmed our assumptions that the tested organic acids disrupted the membrane permeability. Alakomi et al. [48] reported that an increase in the permeability of membranes caused by organic acids enhances the activity of other antimicrobial metabolites. This suggests that the action of organic acids as inhibitors of microbial growth is complex, and other antibacterial agents can be also involved in this process.

In this study, we showed that CFSs derived from *Lactobacillus* isolated from urine exhibited an ability to suppress the growth of *P. mirabilis* bacteria. Interestingly, organic acids were found to be the main factors that displayed these activities. However, further studies are required, concerning their interactions and the influence of other factors such as bacteriocins or hydrogen peroxide on this activity. This study focused on organic acids because they showed the most intensive antimicrobial properties against tested uropathogens. It was revealed that organic acids secreted by *Lactobacillus* have potential in UTI prevention and treatment. These extracellular substances can also have an influence on diseases associated with urinary tract infections such as the development of infectious urinary stones.

## 4. Materials and Methods

### 4.1. Bacterial Strains

*Lactobacillus* strains were obtained from the urinary tract of healthy people. With the consent of the Committee for Bioethics of Scientific Research of the University of Lodz (4/I/2020), sterile urine was collected from volunteers, both men and women, who had not been treated with antibiotics and probiotics in the last 3 months. The samples of urine were centrifuged (1400× *g* for 10 min), the sediments were then seeded on APT agar (BD Difco, Franklin Lakes, NJ, USA) and incubated in microaerophilic conditions in 5% CO₂ at 37 °C for 48 h. Bacteria were isolated and identified from selected colonies. Species confirmation was performed by observing their morphology. Gram-positive and catalase-negative rods were selected for analysis by mass spectrometry MALDI/TOF Microflex LT (Bruker, Billerica, MA, USA). Nine of the 43 isolates were identified as *Lactobacillus* species: *L. crispatus* (n = 6), and one representative each of *L. jensenii, L. gasseri*, and *L. saerimneri*. In addition, reference strains were included in the research: *L. gasseri* ATCC 33323 and *L. crispatus* ATCC 33197.

*Proteus mirabilis* strains were isolated from the urine of individuals with diagnosed infectious urolithiasis (1567; 1090; KP; 5628) by the Department of Microbiology of Children’s Memorial Health Institute in Warsaw, Poland. The other four *P. mirabilis* strains were collected from urinary stones (608/221; K5/MC1; K8/MC; K0) by Provincial Specialist Hospital M. Pirogow in Lodz. The strains were identified using the API 20E test (Biomerieux, Marcy-I’Etoile, France) and were cultured on TSB (tryptic soy broth, BTL, Warsaw, Poland) for 24 h at 37 °C. The susceptibility of these strains to the most common antibiotics used in urinary tract infections treatment was investigated using the method recommended by the European Committee on Antimicrobial Susceptibility Testing (EUCAST). The list of antibiotics used in this assay is provided in Table 1. The diameters of inhibition zones were measured and compared with the EUCAST breakpoints [49]. The results were expressed as the percentage resistance, where 100% was the number of all tested *P. mirabilis* strains (n = 8). All of the isolates were stored at −80 °C as frozen stocks in the presence of DMSO.

### 4.2. Lactobacillus Cell-Free Supernatant (CFS) Preparation

*Lactobacillus* strains were cultured in APT broth at 37 °C for 48 h in 5% CO₂. Subsequently, the cultures were centrifuged (3300× *g*, 4°C, 30 min) and the supernatants were treated by several factors described in well diffusion and broth microdilution assays to evaluate which substances secreted by *Lactobacillus* showed antibacterial properties against tested uropathogens. Obtained supernatants were sterilized by filtration through a sterile syringe filter Minisart (0.22 µm pore size filter, Sartorius, Goettingen, Germany).

### 4.3. Well Diffusion Assay

The agar well diffusion method was used to detect antimicrobial activities of *Lactobacillus* CFSs. These properties were estimated according to the Prabhurajeshwar et al. [50] method with some modifications. The supernatants without the bacteria were separated into two aliquots with the first one left untreated. The second sample was neutralized to pH 6.0 by 1 M NaOH to exclude the impact of organic acids secreted by *Lactobacillus*. *P. mirabilis* strains were cultured on TSB medium for 24 h at 37 °C; afterward, 100 µL of the culture was swabbed on the surface of MH agar plates (Mueller Hinton, Biomaxima, Lublin, Poland). Next, 100 µL of CFS of lactobacilli and 100 µL of APT broth as a negative control were transferred to 7 mm diameter wells. After 24 h of incubation at 37 °C, inhibition zones were measured. 

### 4.4. Broth Microdilution Method

The influence of different substances secreted by *Lactobacillus* was estimated according to Chen et al.’s method [51] with some modifications. The cell-free supernatants (CFSs) of *Lactobacillus* species were separated into four aliquots and used in the broth microdilution method to check the activity of different substances secreted by *Lactobacillus* on *P. mirabilis* growth. The first one was untreated, the second one was neutralized to pH 6.0 by 1 M NaOH to determine the antagonistic effect dependent on organic acids, and the third one was additionally treated with catalase (5 µg/mL, 1 h at 37 °C, Biomerieux, Marcy-I’Etoile, France), to determine if any antibacterial property was due to the presence of hydrogen peroxide. The fourth aliquot was also neutralized to pH 6.0 and heated (70 °C/30 min), which excluded the action of thermolabile proteins such as bacteriocins. Then, 100 µL of *P. mirabilis* suspension in TSB (10^6^ CFU/mL) was added to 96-well plates with 100 µL of CFS of each *Lactobacillus*. The negative control was 100 µL of TSB medium mixed with 100 µL of APT medium. The positive control was 100 µL of bacterial suspension mixed with 100 µL of APT medium. After 24 h at 37 °C of incubation, the inhibition of bacterial growth was detected by measuring turbidity at 600 nm using a microplate reader Multiskan Ex (Labsystems, Helsinki, Finland). The results were expressed as a percentage of *P. mirabilis* growth inhibition, where absorbance equal to the positive control denoted no inhibition and 100% inhibition was the absorbance of the negative control.

### 4.5. Analysis of the Profile of Organic Acids Produced by Lactobacillus 

Samples (untreated CFSs, obtained according to the method described in Section 4.2) were diluted 100,000 times with LC–MS-grade water, filtered through 0.45 µm nylon membrane, and then injected (5 µL) into the 1260 Infinity II LC system coupled with the triple-quadrupole G6470B mass spectrometer system (MS/MS) with Agilent JetStream ionization technology (Agilent Technologies, Waldbronn, Germany). Analytes were separated on the Reprospher 100 C-18 Aqua (100 × 2.0 mm; 1.8 µm) column obtained from Dr. Maisch, High-Performance LC GmbH (Entringen, Germany). The chromatographic separation of the analytes was accomplished at room temperature (25 °C), using gradient elution with the mobile phase consisting of (A) water and methanol (95:5; *v*/*v*) and (B) methanol and water (95:5; *v/v*), both with 0.1% formic acid, delivered at a flow rate of 0.2 mL/min. The analytes were monitored by MS/MS operated in negative electrospray ionization (ESI) mode. Identification and confirmation of the target compounds in real samples were based upon a comparison of retention time and ESI MS spectra with a corresponding set of data obtained by analyzing authentic compounds. A single standard addition method was used to establish levels of citric acid and succinic acid in the study samples (for assay details, see Supplementary Materials).

To determine the concentration of lactic acid in CFSs of *Lactobacillus*, the spectrophotometric method was performed according to Borshchevskaya et al.’s method [52] with some modifications. Briefly, 5 µL of *Lactobacillus* cell-free supernatant was added to 200 µL of a 0.2% solution of iron(III) chloride. The absorbance was measured at 390 nm using a microplate reader Multiskan Ex against the reference solution (200 µL of a 0.2% FeCl_3_ solution). The concentration of lactic acid was expressed in millimoles.

### 4.6. Determination of the Effects of Organic Acids on P. mirabilis Growth Inhibition and Biofilm Formation

The evaluation of the antimicrobial activity of organic acids was performed using the broth microdilution method, with some modifications. First, 100 µL of *P. mirabilis* suspension in TSB (10^6^ CFU/mL) was added to 96-well plates with 100 µL of the three tested organic acids (lactic acid, citric acid, and succinic acid) at different concentrations, as shown in Figure 4. The negative control was 100 µL of TSB medium with 100 µL of organic acids. The positive control was 100 µL of bacterial suspension. After 24 h of incubation at 37 °C, the inhibition of bacterial growth was detected by measuring turbidity at 550 nm using a microplate reader Multiskan Ex. The results were expressed as of the percentage *P. mirabilis* growth inhibition. A biofilm assay was performed on the same plates according to Maszewska et al.’s method [53]. After 24 h of incubation, the biofilms of *P. mirabilis* were washed with 0.85% NaCl to remove unbound cells. To determine the cell viability, the biofilms were treated with 50 µL of MTT (5 mg/mL in PBS, Sigma, St. Louis, MO, USA) and 200 µL of TSB medium. The plate was incubated for 30 min at 37 °C, and then 300 µL of DMSO (dimethyl sulfoxide) and 50 µL of glycine buffer were added to dissolve formazan crystals. Absorbance was measured at 550 nm. The inhibition percentage was calculated by the difference in growth in the absence and presence of organic acids.

### 4.7. The Impact of Organic Acids on P. mirabilis Membrane Integrity

In order to explain the mechanism of action of organic acids and their impact on *P. mirabilis* membrane permeability, the assay with propidium iodide was carried out [54]. First, 500 µL of *P. mirabilis* suspension in TSB (10^6^ CFU/mL) was incubated for 24 h at 37 °C with 500 µL of the appropriate organic acid concentration. The negative control was *P. mirabilis* suspension. After incubation, the samples were centrifuged for 10 min at 14,000× *g*. The obtained sediments were suspended in 1 mL of PBS (phosphate-buffered saline) and centrifuged under the same conditions. Next, the previous step of the procedure was repeated, and 2 µL of propidium iodide (1 mg/mL^−1^ in H_2_O, Life Technologies, Carlsbad, CA, USA) was added; the samples were incubated for 5 min at room temperature in the dark. The residual dye was removed by centrifugation in the above-described conditions and washed once in PBS. Final precipitates were suspended in 200 µL of PBS. The fluorescence intensity was measured using a Spectramax i3 Molecular Devices spectrofluorometer (Syngen Biotech, Wroclaw, Poland) with the following parameters: λex = 535 nm and λem = 617 nm. In addition, the cell density of tested samples was measured at 550 nm using a microplate reader Multiskan Ex, and the fluorescence activity was calculated by dividing the fluorescence results by OD550.

### 4.8. Statistical Analysis

All experiments were carried out at least in triplicate. Statistical analyses were based on t-Student test with the Cochran-Cox adjustment (Cochran’s Q test) and the Mann-Whitney U test performed using Statistica software version 13.3 pl (StatSoft, Krakow, Poland). The results were considered to be statistically significant at *p* < 0.05.

## 5. Conclusions

The microbiota of the urinary tract is still a poorly understood microbiological environment. In this paper, we showed that *Lactobacillus* spp. isolated from urine possess high antibacterial efficacy by inhibiting the growth of the common pathogen *P. mirabilis*, which causes urinary tract infections. This antimicrobial and antibiofilm capacity was mainly attributed to organic acids (lactic, citric, and succinic) secreted by *Lactobacillus*. The obtained results may constitute an important step in understanding the interactions between pathogens and natural microbiota of the urinary tract. Additionally, *Lactobacillus* microorganisms or the organic acids secreted by them could contribute to the prevention or may support the treatment of UTIs in the future. An interesting aspect for further study may be the influence of the natural microbiota of urine and its extracellular substances on UTI complications such as the formation of infectious urinary stones or CAUTIs.

## Figures and Tables

**Table 1:** Antibiotic susceptibility *of P. mirabilis* strains.

Antibiotic Used	Resistant Strains	% of Resistance
Ampicillin	K0; KP; 1090; 1567; 5628; 608/221	75%
Ampicillin/sulbactam	K0; KP; 1090; 1567; 5628; 608/221	75%
Piperacillin/tazobactam	1090; 608/221	25%
Cefepime	1090; 5628; 608/221	38%
Cefotaxime	KP, 1090; 1567; 5628; 608/221	63%
Ceftazidime	KP; 1090; 5628; 608/221	50%
Imipenem	-	0%
Meropenem	KP	13%
Aztreonam	-	0%
Ciprofloxacin	608/221	13%
Norfloxacin	-	0%
Amikacin	KP	13%
Gentamicin	K0; KP; 1090; 1567; 5628; 608/221	75%
Tigecycline	1090; 608/221	25%
Nitrofurantoin	K0; K5/MC1; K8/MC; 1090; 1567	63%

**Table 2:** The percentages of *P. mirabilis* isolate growth inhibition according to broth microdilution method. The results are presented as the mean ± standard deviation (SD) of three experiments. All the results are statistically significant (*p* < 0.05) according to *Cochran’s Q* test.

*P. mirabilis* Strains	1.2	3.2	4	6.2	13.2	22.2	35.3	40	41.4	ATCC 33197	ATCC 33323
K0	98%	98%	95%	79%	97%	73%	97%	97%	97%	98%	100%
KP	97%	98%	95%	74%	96%	64%	96%	96%	97%	98%	100%
K5/MC1	97%	97%	93%	72%	72%	95%	72%	94%	95%	96%	100%
K8/MC	98%	99%	94%	69%	96%	65%	96%	96%	96%	97%	100%
1090	97%	98%	95%	65%	96%	70%	96%	96%	96%	97%	100%
1567	98%	98%	95%	66%	97%	62%	96%	96%	97%	97%	100%
5628	97%	97%	93%	92%	95%	69%	95%	89%	95%	96%	100%
608/221	97%	98%	94%	63%	95%	69%	95%	91%	96%	96%	99%

**Table 3:** The concentration (mM) of organic acids: lactic, citric, and succinic acids in cell-free supernatants of *Lactobacillus* strains. The results are presented as the means of three experiments.

*Lactobacillus* Strains.	Lactic Acid	Citric Acid	Succinic Acid
	Concentration (mM)		
*L. crispatus* 1.2	25.09	-	11.21
*L. saerimneri* 3.2	21.46	-	4.91
*L. crispatus* 4	19.24	-	36.51
*L. crispatus* 6.2	19.24	-	17.12
*L. crispatus* 13.2	17.87	6.37	4.4
*L. jensenii* 22.2	13.32	-	52.11
*L. gasseri* 35.3	10.28	-	0.91
*L. crispatus* 40	12.49	14.12	1.81
*L. crispatus* 41.4	16.06	-	8.51
*L. crispatus* ATCC 33197	9.58	-	4.71
*L. gasseri* ATCC 33323	7.59	11.52	4.61

## Data Availability

Not applicable.

## Supplementary Materials

The following supporting information can be downloaded at: https://www.mdpi.com/article/10.3390/molecules27175557/s1, File S1: LC–MS/MS analysis of the profile of organic acids produced by *Lactobacillus*.
