# Supplementary material for: Organic Acids Secreted by Lactobacillus spp. Isolated from Urine and Their Antimicrobial Activity against Uropathogenic Proteus mirabilis
Source: Molecules. 2022 Aug 29;27(17):5557. doi: 10.3390/molecules27175557 (PMC9457960; doi:10.3390/molecules27175557)
Supplement: Supplementary file 1 [file molecules-27-05557-s001.zip › molecules-1860014-supplementary.pdf]

Supplementary file

Organic Acids Secreted by *Lactobacillus* spp. Isolated from Urine and Their Antimicrobial Activity against Uropathogenic *Proteus mirabilis*

LC–MS/MS Analysis of the Profile of Organic Acids Produced by Lactobacillus

1. Reagents and Materials

All chemicals used throughout this study were commercially available and at least of analytical reagent grade. Acetic acid, propionic acid, butyric acid, lactic acid, succinic acid, and citric acid were from Sigma-Aldrich, (St. Louis, MO, USA). (In)organic solvents and mobile-phase additives suitable for liquid chromatography coupled with mass spectrometry (LC–MS), namely, water, methanol, acetonitrile, and formic acid, were purchased from Merck KGaA (Darmstadt, Germany). Samples were filtered prior to chromatographic analysis using the Agilent Captiva filter vials with a 0.45 μm nylon membrane (Agilent Technologies, Waldbronn, Germany).

2. Instrumentation

An Agilent 1260 Infinity II LC system equipped with a flexible pump integrated with a vacuum degasser model G7104C, temperature-controlled sample storage combined with automated sample injector model G7129C, column compartment model G7116B, and triple0quadrupole G6470B mass spectrometer system (MS/MS) with Agilent JetStream ionization technology (Agilent Technologies, Waldbronn, Germany) was used for the chromatographic experiments. Data acquisition and analysis were performed using the MassHunter software. Analytes were separated on the Reprospher 100 C-18 Aqua (100 × 2.0 mm; 1.8 μm) column from Dr. Maisch, High-Performance LC GmbH (Entringen, Germany). Samples were stored in an ultralow-temperature freezer (Panasonic Healthcare Co., Ltd., Sakata, Japan). For sample shaking, the Multi Speed Vortex MSV-3500 (Biosan, Latvia) was used.

3. Stock Solutions

The stock solutions of citric acid and succinic acid (0.1 M) were prepared daily by dissolving an appropriate amount of powder in LC–MS-grade water. Then, the solutions were kept at 4 °C for no longer than 24 h. The working solutions of all carboxylic acids (analytes) were prepared freshly by dilution of a particular standard solution with LC–MS-grade water as needed and were processed without delay. Importantly, all solutions were stored in airtight glassy vials at 4 °C.

4. Sample Handling

The obtained samples were stored at −80 °C until analysis. The samples were processed without delay, after defrosting at room temperature, using the procedure described in Section 5.

5. Sample Preparation for Chromatographic Analysis

The samples were diluted 100,000 times with LC–MS-grade water followed by filtration through a 0.45 μm nylon membrane, and then placed in a thermostated autosampler set to 4 °C. Then, 5 μL of the resulting solution was injected into the LC–MS/MS system.

6. Chromatographic Conditions

The chromatographic separation of the analytes was accomplished using the Reprospher 100 C-18 Aqua (100 × 2.0 mm; 1.8 μm) column with the mobile phase consisting of (A) water and methanol (95:5; *v*/*v*) with 0.1% formic acid and (B) methanol and water (95:5; *v*/*v*) with 0.1% formic acid, delivered at a flow rate of 0.2 mL/min. The chromatographic separation was performed at room temperature (25 °C) using gradient elution: 0–4 min, 100–80% A; 4–6 min, 80–100% A; 6–12.5 min, 100% A. The MS/MS detector was operated in negative electrospray ionization (ESI) mode with MRM optimization and ion source optimization, including the LC flow-dependent and compound(s)-dependent parameters of ion source optimization, conducted using the MassHunter Optimizer and Ion Source Optimization Source and iFunnel Optimizer.

7. Qualitative Analysis

The identification and confirmation of the target compounds were performed by analyzing the standard solution of carboxylic acid (100 nM) processed according to the procedure described in sections 5 and 6. Initially, the analyses were carried out using the LC–MS/MS system, which was set to acquire data in scan mode. The ESI MS spectra were recorded by setting the instrument to gather data, stepping the mass filter within the *m*/*z* 30–450 range. In all cases, products with a deprotonated molecular ion $[M-H]^-$ were observed. The confirmation of the origin of the particular peak of the compound of interest in real samples was based upon a comparison of retention time and specific ESI MS spectra with a corresponding set of data obtained by analyzing an authentic compound. In order to increase sensitivity and selectivity in the trace analysis, analyses were also conducted with SIM and MRM MS modes to confirm or exclude the presence of a particular carboxylic acid in study samples.

8. Quantitative Analysis

A single standard addition method was used to establish levels of citric acid and succinic acid in the study samples. For this purpose, the analyses were conducted in MRM MS mode. The target compounds were quantified using the precursor to product ion transitions of *m*/*z* 191.1 → 111.0 and *m*/*z* 117.1 → 73.2 for citric acid and succinic acid, respectively. The endogenous concentrations of the analyte(s) were evaluated on the basis of the data obtained from triplicate analysis of a particular blank sample from an individual source and the same sample spiked with a known quantity of the analyte(s), providing a concentration in the study sample of 100 nM. The concentration of the analyte(s) in each sample was calculated using the following formula:

$$C_x = \frac{Y_x \times C_s}{Y_s - Y_x},$$

where C_x is the concentration of the analyte in the blank sample, Y_x is the analytical signal for the sample containing only the analyte, C_s is the concentration for the sample with the addition of a known amount of the standard, and Y_s is the analytical signal for the sample with the addition of a known amount of the standard.
